# Material Removal Rate Enhancement Induced by Electrochemical Discharge Machining for Refractory High-Entropy Alloys Compared with EDM

**DOI:** 10.3390/e27090912

**Published:** 2025-08-29

**Authors:** Bolin Dong, Zirui Yao, Chen Qi, Xiaokang Yue, Zufang Zhang, Shunhua Chen

**Affiliations:** 1 School of Mechanical Engineering, Hefei University of Technology, Hefei 230009, China; bolin.dong@hfut.edu.cn (B.D.); 2023110127@mail.hfut.edu.cn (Z.Y.); 77qc@xy.hfut.edu.cn (C.Q.); xkyue@hfut.edu.cn (X.Y.); zufangzh@hfut.edu.cn (Z.Z.); 2 Key Laboratory of Advanced Functional Materials and Devices of Anhui Province, Hefei University of Technology, Hefei 230009, China

**Keywords:** refractory high-entropy alloy, electrochemical discharge machining, electric discharge machining, material removal rate, surface quality

## Abstract

Refractory high-entropy alloys (RHEAs) are categorized as difficult-to-machine materials due to their excellent mechanical properties. Electrical discharge machining (EDM) is a special processing method for RHEAs, which faces challenges such as low machining efficiency. In this work, electrochemical discharge machining (ECDM) was proposed for (TiVZrTaW)_99.5_N_0.5_ and (TiVZrTa)W_5_ (at. %, denoted as W20N0.5 and W5, respectively) RHEAs, and their machining performances were investigated and compared with EDM. At a peak current of 25 A, the material removal rate (*MRR*) using ECDM is more than twice that of EDM for W20N0.5 (reaching to 1.24 mm^3^/min) and 1.5 times higher than that for W5. Both W20N0.5 and W5 RHEAs exhibited higher *MRR* in ECDM based on the analyses of the influence of top diameter, bottom diameter, machining depth, and surface roughness (*R*a). The process and mechanisms of material removal were examined through the microstructural morphology and elemental distribution analyses. This work proposed a more effective route for machining RHEAs by ECDM compared to the conventional EDM.

## 1. Introduction

In 2010, Senkov et al. [1] introduced the Mo, Ti, V, Nb, Hf, Ta, Cr, and W refractory elements and put forward the concept of “Refractory High-entropy Alloys” (RHEAs). Recently, RHEAs have inherited and overwhelmed traditional alloys due to their impressive mechanical properties, such as superior hardness, high strength, great wear resistance, extremely corrosion resistance, and superior thermostability [2,3,4,5,6,7]. RHEAs have become emerging advanced materials and can be applied in new fields like thermoelectric, magnetocaloric, superconducting, and catalysis materials [8]. However, RHEAs are also classified as difficult-to-machine materials due to their exceptional mechanical properties [9]. Electrical discharge machining (EDM) is a non-traditional machining technique used to machine conductive materials with high hardness and melting point, such as titanium alloys [10,11,12,13,14,15,16], Ni-based alloys [17,18], metal matrix composites [19], and bulk metallic glasses (BMGs) [20,21,22].

Recently, efforts have been made to investigate the wire electrical discharge machining (WEDM) of RHEAs. The results have shown that during WEDM, the cutting efficiency (CE) among the different materials is mainly affected by the differences in the melting point [23], and the microcracks, voids, globules, craters, and debris are formed on the surfaces when machining MoNbTaTiZr [24] and HfNbTaTiZr [25]. For RHEAs with high melting points, it is still challenging to improve machining efficiency by discharge machining. Furthermore, the presence of multi-phase structures or higher content of additional phases is conducive to the removal of materials by establishing the relationship between the constituent phase and processing parameter [26]. Fortunately, a more recent work has confirmed the superior, controllable sinking EDM performance of W-containing RHEAs [27]. The results have shown that the sinking EDM technique is suitable for the processing of RHEAs. However, low machining efficiency is still worthy of further investigations but challenging due to its high melting points that impede the removal of materials by sparking.

Electrochemical discharge machining (ECDM) is a hybrid process combining EDM and electrochemical machining (ECM) [28], where electrochemical anode dissolution and electric discharge erosion occur simultaneously in the machining area. It can be used to machine electrically conductive and non-conductive materials. For different workpiece materials, the ECDM and its variants were successively proposed [29,30,31,32,33]. For RHEAs with excellent corrosion resistance, how to achieve their machinability while ensuring machining efficiency is still an issue to be solved. Utilizing typical electrolytic dielectrics, Ahmed et al. [34] proposed a hybrid method of EDM and ECM, in which the defective recast layer is eliminated and the material removal rate (*MRR*) is maintained simultaneously. However, few studies have focused on the ECDM of RHEAs.

In this work, two RHEAs with different corrosion resistance, i.e., (TiVZrTa)W_5_ and (TiVZrTaW)_99.5_N_0.5_ RHEAs, were selected as workpiece materials. An ECDM technique was proposed to machine blind holes compared with conventional EDM, aiming to improve machining efficiency. The top diameter, bottom diameter, machining depth, and slope of holes were chosen as geometric characteristics, where the *MRR* was calculated and discussed. Furthermore, microstructural morphology and elemental characterizations were performed to reveal the material removal mechanisms.

## 2. Materials and Methods

### 2.1. Preparation of RHEAs

(TiVZrTaW)_99.5_N_0.5_ and (TiVZrTa)W_5_ RHEAs (at. %, denoted as W20N0.5 and W5, respectively) were used as workpiece materials for a comparative study. All samples were prepared by vacuum arc melting using high-purity materials (>99.95 wt.%) under a high-purity argon atmosphere in a water-cooled copper crucible. To ensure chemical homogeneity, the ingots were remelted 12 times. Sample sizes of 5 mm × 10 mm × 0.6 mm were used for ECDM and EDM. For electrochemical testing, 3 mm × 3 mm × 6 mm specimens were prepared through grinding with silicon carbide papers (P400 to P2000 grits), followed by mechanical polishing using a SiO_2_ suspension. An epoxy resin encapsulation was applied to define an exposed electrochemical testing area of 0.09 mm^2^.

### 2.2. Electrochemical Measurements

All the electrochemical tests were performed in a 3.5 wt.% NaCl solution at 25 °C under atmospheric pressure. All the measurements were carried out on an electrochemical workstation (DH7000C, DONGHUA, Nanjing, China) with a standard three-electrode cell system, where the saturated calomel electrode (SCE) was used as the reference electrode (RE), the Pt sheet was used as the counter electrode, and the RHEA was used as the working electrode (WE). To obtain a steady potential value, the open-circuit potential (OCP) was measured for 1800s before each electrochemical test. Tafel polarization curve tests were conducted over the potential range from −0.5 V to 3 V (vs SCE) at a scan rate of 5 mV/s.

### 2.3. ECDM and EDM Setup

**Figure 1:**
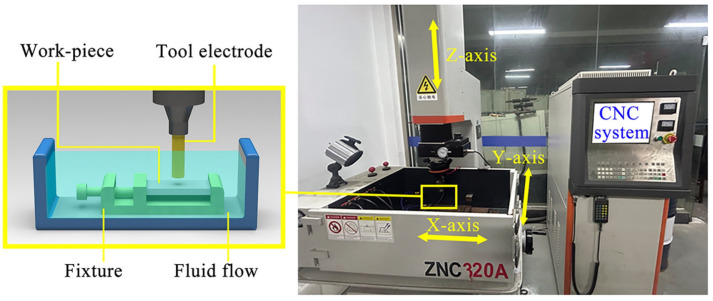
ZNC 320 A machining tool.

All experiments were carried out on a ZNC320A (EXCELLENT, Suqian, China) single-spindle EDM machine tool (Figure 1) equipped with a three-axis linear stage and a precisely controlled motion system. The ECDM was carried out on two RHEAs with different characteristics by machining blind holes. The workpiece was fixed in a circulating tank of dielectric fluid, and a servo control system was used to strictly control the downward feed of the tool electrode and to maintain a constant spark gap. The input parameters required for comparative tests are listed in Table 1. Positive polarity machining mode was adopted. A copper rod with a diameter of 2 mm was selected as the cathode electrode. In order to avoid the occurrence of short circuit phenomenon and the decrease in EDM zone during ECDM [34], the 20 mg/L NaCl solution with deionized water was selected as the working fluid for ECDM. The commercial EDM oil was used for EDM. Additionally, in our preliminary results, *I*p had a larger effect on *MRR* than other parameters such as pulse width and pulse interval. As the core parameter in electrical machining, *I*p directly determines the intensity of single-pulse discharge energy and plays a decisive role in the machining performance [23]. Therefore, peak current *I*p was chosen as the single factor variable here, at four levels: 10 A, 15 A, 20 A and 25 A. Typically, the electrical conductivity of the NaCl solution and EDM oil was 58.35 μS/cm and 0.064 μS/cm, respectively. The experiment was repeated three times for each set of machining parameters to ensure the accuracy of the machining data.

### 2.4. Microstructure and Element Characterization

The surface morphologies, including the bottoms and sides of machined blind holes by ECDM and EDM, were characterized on a scanning electron microscope (SEM) (SU8020, Hitachi, Tokyo, Japan). As a technique for elemental characterizations, energy dispersive spectrometer (EDS) has been widely used in the literature to characterize the elemental distributions, including EDMed surfaces [3,21,23,27]. Here, the chemical composition and elemental distribution were also analyzed using EDS. To minimize the errors in EDS elemental scanning caused by rough surface of the recast layer, map scanning was employed instead of point scanning. Moreover, SEM images at different magnifications were both taken to provide a more detailed comparison. The white light interferometry (Super View W, CHOTEST, Guangdong, China) was used to measure surface roughness.

## 3. Results and Discussion

### 3.1. Materials Properties of W20N0.5 and W5

**Figure 2:**
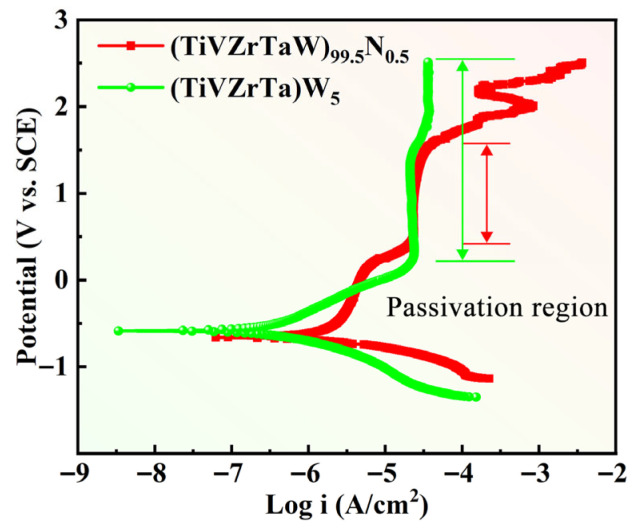
The polarization curve of the W20N0.5 and W5 RHEAs in 3.5 wt.% NaCl solution.

Figure 2 shows the Tafel curves of the W20N0.5 and W5 RHEAs in a 3.5 wt.% NaCl solution at 25 °C. The corrosion potential (*E*corr), corrosion current density (*I*corr), transpassive potential (*E*t) and passivation potential (*ΔE*) obtained from the Tafel curves are listed in Table 2. It is noteworthy that the W5 alloy exhibits a high transpassive potential (*E*t) over 2.5 V_SCE_. W5 RHEA has a wider passivation region and lower *I*corr compared to W20N0.5 RHEA. W20N0.5 has higher hardness and strength but lower corrosion resistance than W5. It can be seen that the two RHEAs both have relatively high hardness and yield strength, which can be classified as difficult-to-machine materials. The melting points are calculated theoretically according to empirical parameters, which will be used to explain the mechanism of EDM. The detailed properties are listed in Table 2.

### 3.2. Machining Efficiency of W20N0.5 and W5

The machining efficiency of blind holes was characterized by the material removal rate (*MRR*), defined as the volume of material removed per unit time, commonly expressed in mm^3^/min. The *MRR* was determined by the following equation:

(1) MRR=V/t

where *V* is the volume of the removed materials (mm^3^) and *t* is the machining time (min).

**Figure 3:**
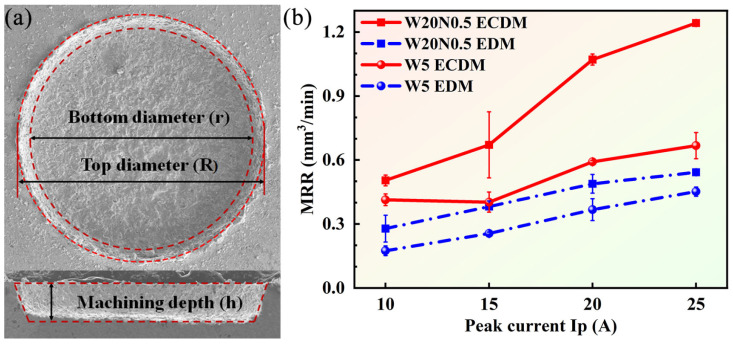
(**a**) Schematic diagram of parameter selection; (**b**) effects of peak current on *MRR*.

An image-based measurement approach was adopted to calculate the volume of the material removed. To ensure measurement accuracy, the processing time was strictly controlled, and three repeated experiments were conducted for each parameter set to guarantee computational reliability. Figure 3a shows a specific example diagram of parameter measurement. *h* denotes the depth of the blind holes (mm), *R* is the top diameter (mm), and *r* presents the bottom surface diameter (mm).

The variations in *MRR* for W20N0.5 and W5 under different peak currents are shown in Figure 3b. It can be found that the *MRR* increased gradually with the increment of *I*p regardless of the workpiece material and machining techniques. The increased *I*p caused an increase in discharge energy [24], which facilitated the melting and vaporization of workpieces. For W20N0.5, the difference value in *MRR* between ECDM and EDM increased with *I*p increasing. It should be pointed out that since ECDM is a hybrid process combining EDM and ECM, it was still challenging to quantify the synergistic effect of pure ECM in ECDM. Here, we mainly compared the *MRR* of ECDM with pure EDM. Nevertheless, it would still be interesting to quantify the synergistic effect of the two mechanisms (ECDM and pure ECM) in the future to give more insight into the underlying mechanisms of the ECDM. Specifically, at 25 A, the *MRR* by ECDM was more than twice that of EDM, up to 1.24 mm^3^/min. For W5, the difference value in *MRR* between ECDM and EDM remained almost stable with the increase in *I*p, and the *MRR* in ECDM was 1.5 times higher than in EDM. For EDM, the workpiece material is mainly removed through melting and vaporization. However, for ECDM, by introducing the electrochemical effect, the *MRR* using ECDM was always higher than that using EDM, regardless of the two RHEAs. Moreover, the RHEAs with different corrosion resistance levels had different efficiency for material removal, where the W20N0.5 alloy exhibited much higher *MRR* than the W5 alloy.

### 3.3. Effects of Peak Current on the Hole Shape

**Figure 4:**
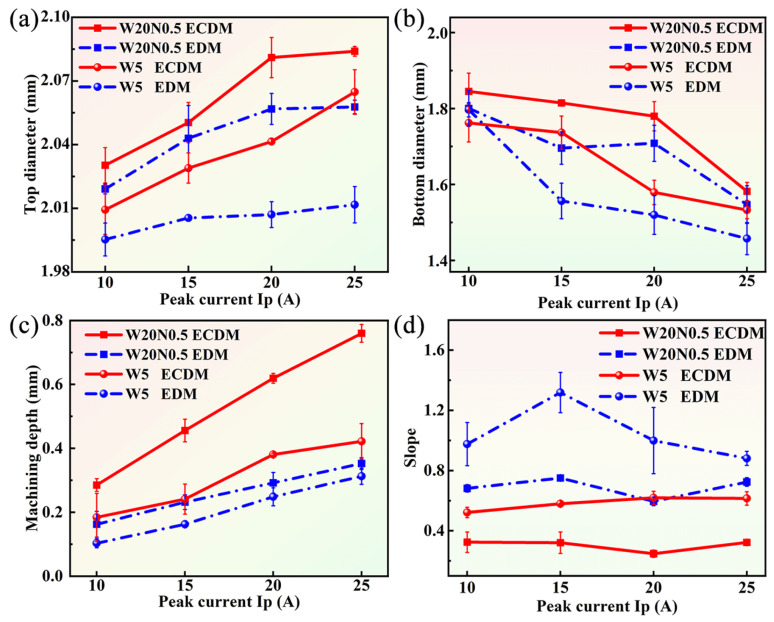
Effect of peak current on the (**a**) top diameter, (**b**) bottom diameter, (**c**) machining depth, and (**d**) slope.

The hole shape was represented by the top diameter, bottom diameter, machining depth, and hole slope. The comparison of hole shape for W20N0.5 and W5 under different *I*ps is given in Figure 4. For two RHEAs, regardless of ECDM and EDM, the top diameter increased as the *I*p increased from 10 A to 25 A. The increase in *I*p led to an increase in discharge energy. Conversely, the bottom diameter decreased with increasing *I*p due to the wear of the tool electrodes. Figure 4c shows the effect of *I*p on the machined hole depth, where the machined depth increased with increasing *I*p. The current density of the bottom sidewalls increased with the decrease in machining gap, which led to an increase in the diameter of the bottom surface using ECDM. As a result, the slope of ECDM was smaller due to the occurrence of electrochemical dissolution along the sidewalls of the machined blind holes, as shown in Figure 4d.

### 3.4. Morphology of Holes at Lower Peak Current

**Figure 5:**
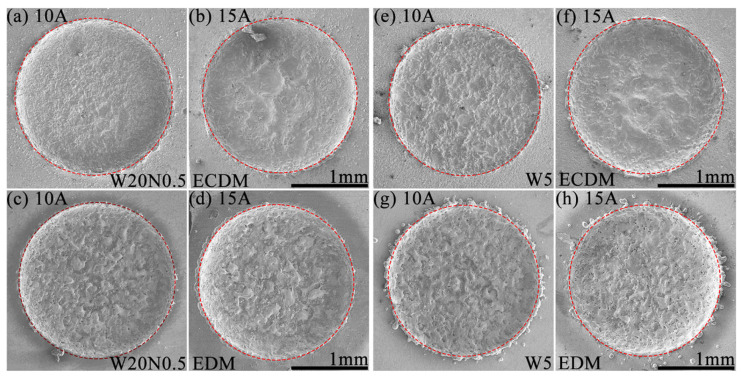
SEM images of blind holes machined at different *I*p 10 A,15 A: (**a**–**d**) W20N0.5, (**e**–**h**) W5.

Figure 5 shows the comparison of machined surfaces of two RHEAs obtained from EDM and ECDM. Overall, the RHEA machined by ECDM (Figure 5a,b,e,f) showed smoother hole edges and bottom surfaces, while the EDMed holes (Figure 5c,d,g,h) demonstrated rougher craters and more debris due to the low melting point and viscosity of the melted liquids. More specifically, at 10 A, the W20N0.5 alloy showed a flatter machined surface with small craters in ECDM (Figure 5a) compared with that in EDM (Figure 5c). As the *I*p increased to 15 A (Figure 5b), larger discharge craters with smoother surfaces were generated for this alloy in ECDM, while the EDMed surfaces exhibited a messy recast layer. For the W5 alloy with better corrosion resistance, the surfaces after ECDM (Figure 5e,f) were smoother than those of EDM but rougher than those of the W20N0.5 alloy. This may be related to the different corrosion resistance levels of RHEAs, as the electrochemical effect responsible material removal was diminished in the more corrosion-resistant W5 alloy.

### 3.5. Sidewall Surface Characteristics of Machined Blind Holes by ECDM and EDM

**Figure 6:**
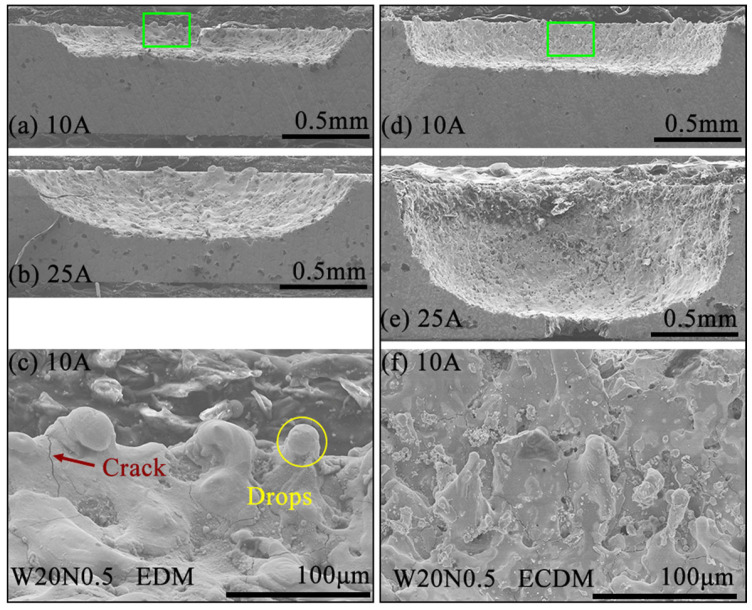
SEM images of W20N0.5 RHEA (side surface) machined by EDM (**a**–**c**) and ECDM (**d**–**f**) at *I*p 10 A and 25 A. The rectangle regions of (**a**,**d**) are further magnified and displayed in (**c**,**f**), respectively.

The sidewall surface characteristics of W20N0.5 RHEA processed by ECDM and EDM at different peak currents (10 A and 25 A) are shown in Figure 6. The rectangle regions in Figure 6a,d are further magnified and displayed in Figure 6c,f, respectively, for a closer observation of the surface characteristics. It can be seen that the machined depth in ECDM was significantly deeper than in EDM. For W20N0.5 RHEA, the machined hole became deeper with increased *I*p. The enhancement in depth using ECDM in Figure 6e was more evident than that in EDM in Figure 6b.

On the other hand, side morphology shows the slope of the machined holes. The higher value of peak current caused more severe tool wear. As *I*p increased to 25 A, the severe tool wear caused arc-shaped side surface for EDM. In addition, molten materials cooled down in a short time, and stress was generated, causing the formation of cracks and drops on the machined surface (in Figure 6c). For ECDM, the molten materials remained on the workpiece but with much smaller sizes, indicating that more molten materials were removed during the feeding of tool electrode. Under the electrochemical corrosion and dissolution, the surface quality of the side surface became better in ECDM (Figure 6f) compared to that in EDM (Figure 6c).

**Figure 7:**
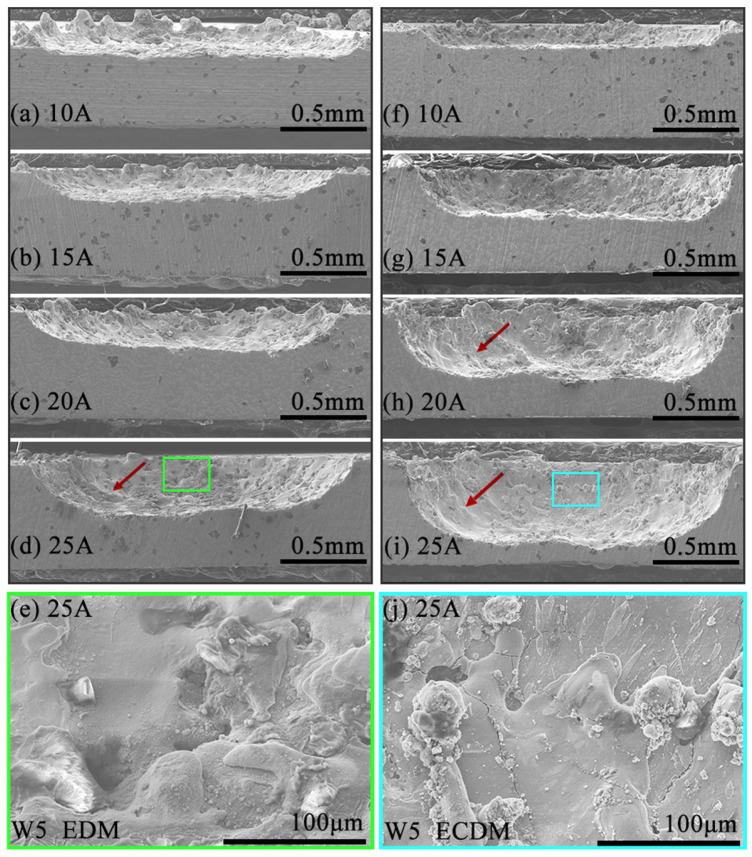
SEM images of W5 RHEA (side surface) machined by EDM (**a**–**e**) and ECDM (**f**–**j**). The rectangle regions of (**d**,**i**) are further magnified and displayed in (**e**,**j**), respectively.

Figure 7 shows the W5 RHEA side surface morphology of machined blind holes by EDM and ECDM. Compared to W20N0.5 RHEA, the W5 RHEA was more difficult to be removed using EDM and had relatively lower machining depth (Figure 7a–d). During the EDM process (Figure 7e), some molten material remained on the workpiece and subsequently resolidified during the cooling process, forming a recast layer. The hole depth increased with the increase in *I*p. Using ECDM, the machined depth of W5 RHEA was improved compared to that from EDM, as shown in Figure 7f–i. However, due to the higher corrosion resistance, the electrochemical dissolution was prevented for W5 RHEA in ECDM, resulting in smaller hole depth compared with that in Figure 6e.

### 3.6. Machined Bottom Surfaces and Surface Roughness of RHEAs

**Figure 8:**
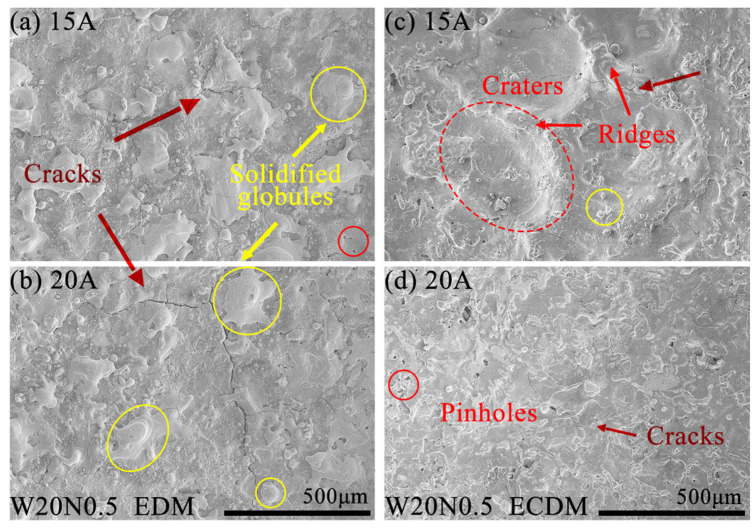
SEM images of machined bottom surfaces for W20N0.5 RHEA at *I*p of 15 A and 20 A (**a**,**b**) using EDM and (**c**,**d**) using ECDM.

Figure 8 depicts the surface morphology of W20N0.5 using EDM and ECDM with *I*p of 15 A and 20 A. During EDM, as shown in Figure 8a,b, small, solidified globules and cracks distributed randomly on the EDMed surface. The molten material re-solidified on the machined surface after rapid cooling, forming globules of debris. The cracks observed on the machined surfaces, resulting from rapid cooling by the dielectric fluid during the pulse off time following each tool electrode feeding [35]. This rapid cooling induced residual tensile stress. As *I*p increased, the cracks became longer and wider, connecting to the edge of solidified globules, as seen in Figure 8b.

Compared to EDM, the ECDMed machined surfaces (Figure 8c,d) had different morphology features, including electrochemical craters, microcracks and pinholes. At *I*p of 15 A, as shown in Figure 8c, ridges were observed around the craters instead of solidified globules in EDM. As *I*p increased to 20 A, electrochemical dissolution played the leading role in removing the remelted materials. It can be seen that the craters with ridges were dissolved into a relatively flat machined surface, resulting in a better surface quality for the W20N0.5 alloy in ECDM.

**Figure 9:**
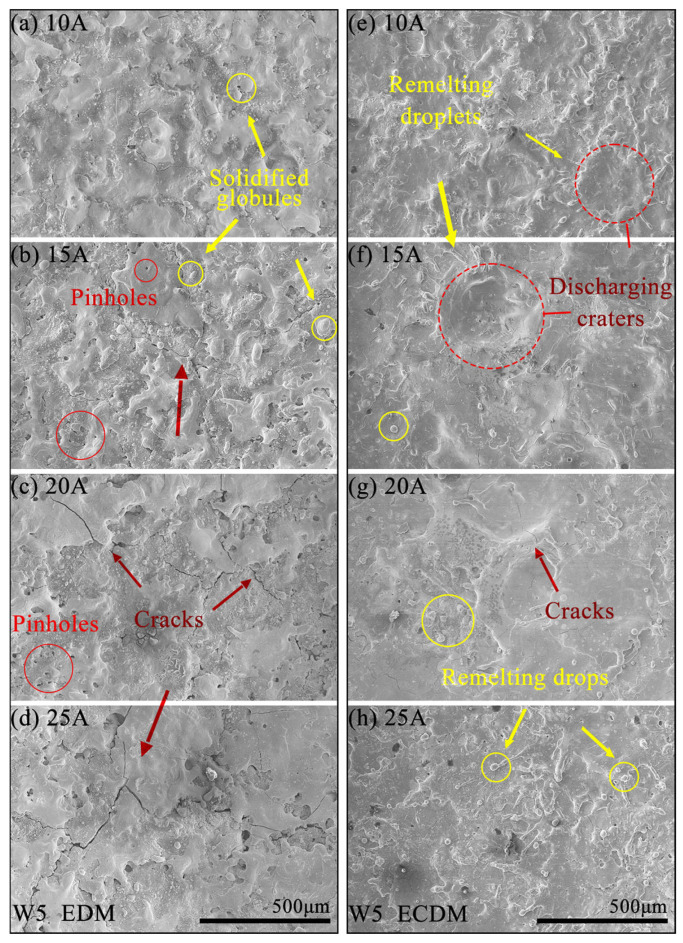
SEM images of machined bottom surfaces for W5 RHEA at different *I*p from 10 A to 25 A: (**a**–**d**) EDM, (**e**–**h**) ECDM.

Figure 9 shows the surface for W5 using EDM and ECDM at different *I*ps. All SEM images were observed at the center of machined surfaces. Figure 9a–d depicts the influence of *I*p on the machined bottom surfaces processed by EDM. As *I*p increased, circular solidified globules and pinholes were observed on the machined surface in Figure 9a,b. This may be due to the uniform heat generated during the EDM process. Higher discharge energy intensified the recast layer on the machined surface due to the increase in *I*p and significantly increased the number of cracks (Figure 9c,d).

As shown in Figure 9e–h, the ECDMed surfaces of the W5 alloy exhibited distinct characteristics compared to that of EDM. Figure 9e shows that discharge craters were concentrated around the center of the machined surface. The bottom of the craters was flatter than other areas, but the edge of the craters consisted of remelting droplets in Figure 9e,f. The reason for that was the uneven current density generated by the machining gap during the ECDM process. A larger current density can accelerate the electrochemical dissolution of materials, leading to flatter crater bottom, while the lower current density slowed the material dissolution rate to form droplets.

Additionally, a few small, solidified globules and microcracks still randomly distributed on the machined surface by ECDM. At the maximum *I*p of 25 A (Figure 9h), there was no discharge craters on the machined surface, but remelting drops remained. Increased discharge energy reduced the surface finish quality compared to 20 A (Figure 9g). Overall, compared with the bottom surface characteristics after EDM, ECDM significantly improved machining surface quality and greatly increased machining efficiency.

**Figure 10:**
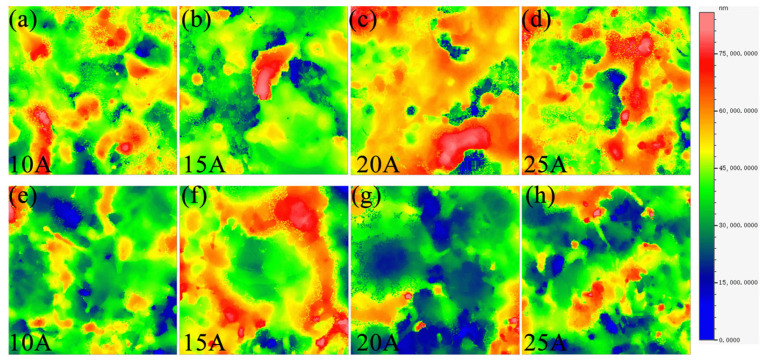
Three-dimensional morphology of bottom-machined surfaces for the W5 alloy at different *I*ps: (**a**–**d**) in EDM and (**e**–**h**) in ECDM.

In order to further analyze the morphology of the bottom surfaces of machined blind holes, three-dimensional morphological evolution is shown in Figure 10. The intermediate region (red area) exhibited solidified protuberances with higher *R*a in Figure 10a–d, which was primarily attributed to the uneven released heat during EDM. As *I*p increased, the uneven released heat of EDM was more serious, resulting in more protrusion features, as shown in Figure 10c,d.

**Figure 11:**
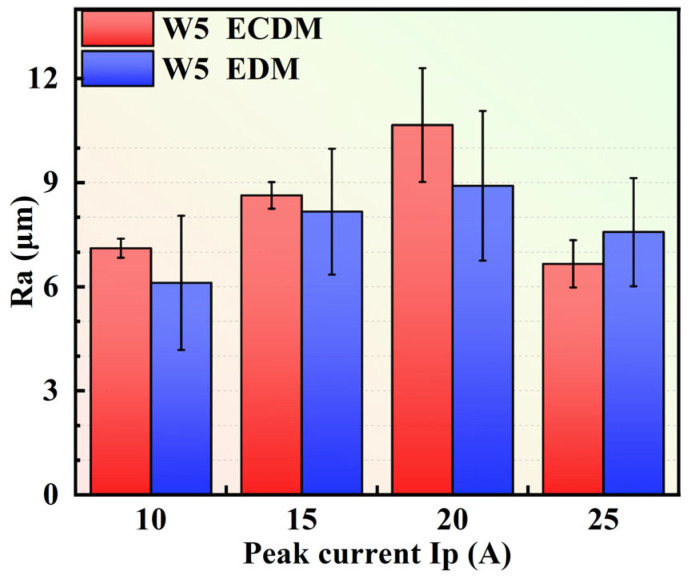
The *R*a of bottom-machined surfaces on W5 alloy during ECDM and EDM at different *I*ps.

During ECDM, it had a different evolution of characteristics. As *I*p increased from 10 A to 15 A, the small-sized craters changed into volcanic-like craters whose edge consisted of ridges (red area) in Figure 10f. At 20 A, a higher discharge energy accelerated the materials’ removal on the ridges of the craters. Increasing *I*p enhanced the effect of electrochemical dissolution in ECDM, thereby improving the surface quality on machined surfaces in Figure 10g,h. As shown in Figure 11, at the *I*p of 20 A, the ECDMed surface showed increased *R*a compared to the results of the EDMed (at 20 A) and the ECDM at other current values. From 10 A to 20 A, the *R*a produced by EDM was smaller than ECDM because of the excellent corrosion resistance of the W5 alloy. However, the ECDMed results have smaller errors compared to the EDMed results, indicating a more stable material removal process.

**Figure 12:**
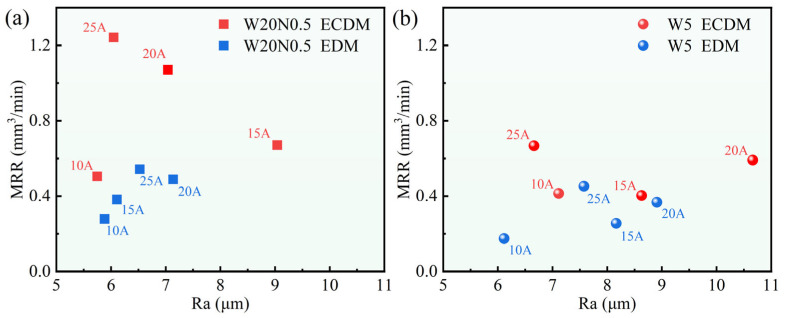
The distribution of average *MRR* and *R*a data of W20N0.5 (**a**) and W5 (**b**) using ECDM and EDM.

Figure 12 shows the relationship between *MRR* and *R*a for different machining methods. For the W20N0.5 alloy in Figure 12a, which has a relatively worse corrosion resistance, *I*p significantly influenced *R*a. As *I*p increased from 15 A to 25 A, *MRR* increased while *R*a decreased in ECDM. At the *I*p of 25 A, it was able to achieve the lowest *R*a and the highest *MRR* for W20N0.5 alloy. However, increased *I*p did not have a more significant effect on *MRR* and *R*a in EDM compared to ECDM. This was mainly attributed to the fact that, in EDM, the workpiece material was removed by melting and expulsion of molten materials, whereas in ECDM, electrochemical dissolution also contributes to the removal process.

In Figure 12b, the *MRR* of the W5 alloy was enhanced more significantly at the *I*p, from 20 A to 25 A. At the same time, the surface quality was improved at 25 A. In contrast, the *MRR* of W5 using ECDM was lower than W20N0.5 using ECDM, owing to the better corrosion resistance of the W5 alloy. The reports on the WEDM of RHEAs have shown that it is still challenging to improve machining efficiency and surface quality [23,24]. The present ECDM provided a new hybrid machining way, and both the machining efficiency and surface quality can be improved by introducing ECM to EDM. With the same basic discharging machining mechanisms, further introducing ECM in WEDM may still be useful for improving its machining performance. Furthermore, EDM optimization methods, such as the optimization of parameters by establishing mathematical models [27], were usually based on certain workpiece materials or certain parameter groups. Here, introducing ECM to EDM changes the basic processing mechanisms, which could also be applicable for some other similar workpiece materials and parameters. To sum up, it can be concluded that the ECDM was a more effective route for machining RHEAs compared to the conventional EDM, and such mechanism may also be applied to other situations, including WEDM, to improve machining performance.

### 3.7. Material Removal Process and Mechanism

**Figure 13:**
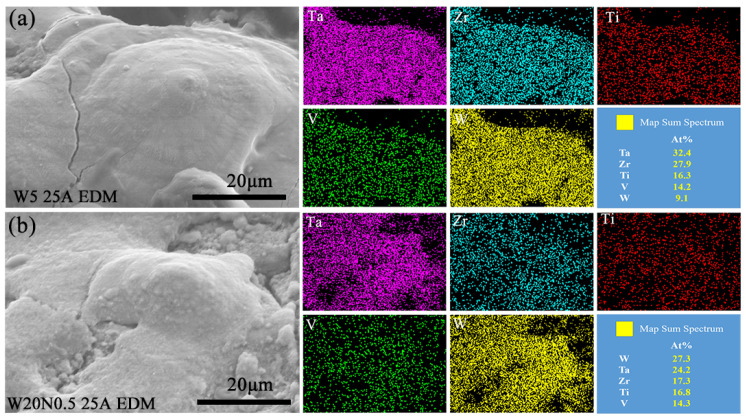
Elemental mapping images of the molten protuberance on the bottom surfaces by EDM: (**a**) W5 alloy and (**b**) W20N0.5 alloy.

During EDM at 25 A, the materials with high melting points that were not removed underwent remelting and adhered to the machined surface, forming protrusion structures. Both Ta and W were mainly concentrated in the recast layer of the molten protuberance, as shown in Figure 13. According to the results of EDS scanning, the removal of elements with low melting points, such as Ti and V, was more significant in EDM. Differences in melting points led to varied removal behavior among the constituent elements, with significant depletion observed for elements of lower melting points. According to the content of matrix elements, Ta and W with high melting points also enriched the recast surface during the EDM of W20N0.5 RHEA.

**Figure 14:**
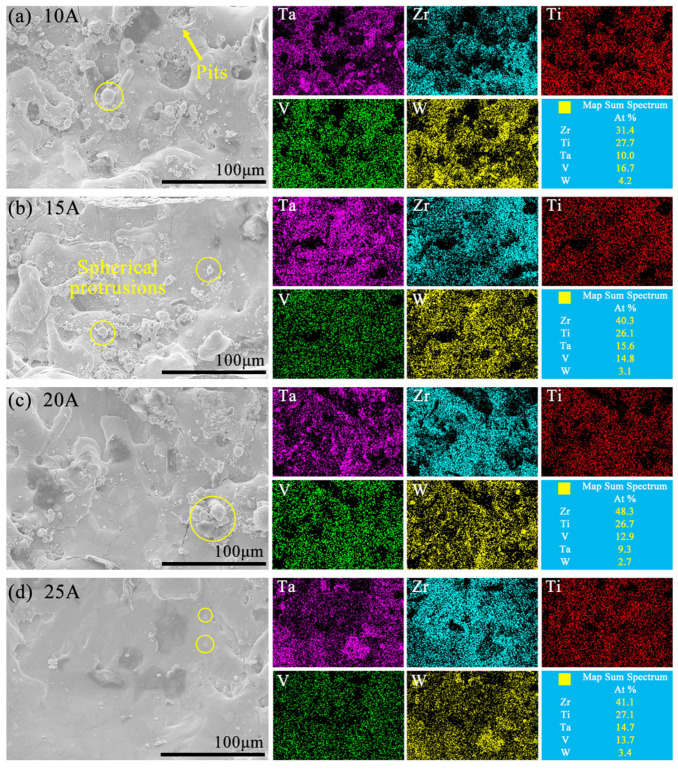
Elemental mapping images on the sidewall of machined holes of W5 RHEA by ECDM using different *I*p from 10A to 25A (**a**–**d**).

Figure 14 displays the surface morphology and EDS results of the machined sidewall for the W5 alloy by ECDM. After ECDM, it can be seen that the elements of the machined surfaces were changed, as the proportions of Zr and Ti increased significantly. At 10 A (Figure 14a), the sidewall morphology exhibited pits and spherical protrusions. As *I*p increased, enhanced discharge energy promoted continuous electrochemical reactions, gradually flattening the surface. At 25 A (Figure 14d), superior sidewall surface quality was achieved while maintaining the surface enrichment of Zr and Ti and the deficiency of W. It is because W had the highest standard electrode potential, as shown in Table 3. The effect and priority of participating in electrochemical dissolution were the lowest. The other elements, such as Zr and Ti, had lower standard electrode potentials, which made it easier to form corresponding oxide in ECDM.

**Figure 15:**
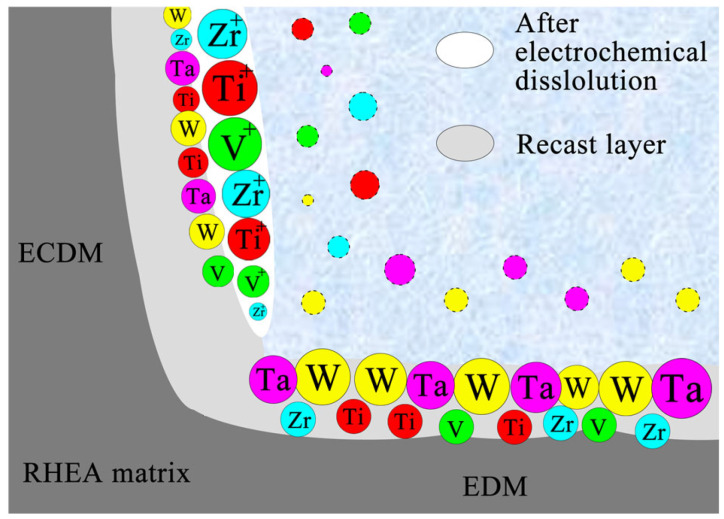
Schematic diagram of the material removal mechanism of RHEA.

Based on above, elements with low melting points, such as Ti and V, were more prone to melting and continuous removal during the generation of bubbles and the release of heat from sparking during EDM. In contrast, the remaining material formed protrusions rich in Ta and W, eventually creating a recast layer, as shown in Figure 15.

However, the elemental composition of the surface after ECDM in the NaCl solution exhibited the opposite trend to that observed in EDM. The amount of Zr and Ti on the machined surface was relatively higher compared to the original RHEA matrix material. This phenomenon may be attributed to the following two primary reasons. On the one hand, the (TiVZrTa)W_5_ RHEA contained both a main phase and a secondary phase, similarly to the W_15_ (TaVZr)_85_ RHEA. During the EDM stage of ECDM, the secondary phase enriched in Zr and Ti preferentially migrated to the outer recast layer due to element migration mechanisms [36]. On the other hand, the standard electrode potentials of Zr and Ti were lower that those of other elements (as shown in Table 3). Their lower potentials made Zr and Ti more prone to forming an oxide-passive film on the surface, which corresponded to excellent corrosion resistance. After ECDM, this also led to enrichment of Zr and Ti—elements with lower melting points— on the machined surface, as illustrated in Figure 14.

## 4. Conclusions

In this work, the machining efficiency of W20N0.5 and W5 RHEAs by ECDM, compared with conventional EDM, was analyzed. The main findings are concluded as follows:

1. The W5 alloy exhibited better corrosion resistance than W20N0.5, as indicated by an *I*corr 1.133 × 10^−7^ A/cm^2^, a wider passive zone, and a transpassive potential above 2.5 V_SCE_. However, the W20N0.5 alloy showed superior mechanical properties, including higher hardness and strength. These differences in corrosion resistance resulted in different *MRR* performance during ECDM.
2. Based on single-factor experiments, the difference in *MRR* between EDM and ECDM increased with the increment of *I*p. The *MRR* of the W5 alloy was lower than that of the W20N0.5 alloy due to the excellent corrosion resistance of W5. Nevertheless, the findings confirm that, compared with conventional EDM, significantly higher *MRR* can be achieved in RHEAs by using ECDM.
3. The EDS results showed that elements with high melting points (Ta and W) enriched at the bottom of the machined surface after EDM. In contrast, elements with low melting points (Zr and Ti) enriched at the machined surface after ECDM.

## Figures and Tables

**Table 1:** Input process parameters for ECDM and EDM.

Parameters	Machining Methods	
	ECDM	EDM
Work fluids	NaCl solution	EDM oil
Electrical conductivity	58.35 μS/cm	0.064 μS/cm
Tool electrode	Copper electrode	
Gap voltage *V*g	30 V	
Pulse duration *T*on	30 μs	
Pulse interval *T*off	80 μs	
Machining time T	100 s	
Peak current *I*p	10, 15, 20, 25 A	

**Table 2:** Material properties of (TiVZrTaW)_99.5_N_0.5_ and (TiVZrTa)W_5_ RHEAs.

RHEAs	Properties						
	*T*m (K)	Hardness (HV)	*σ*_y_ (MPa)	*I*corr (A/cm^2^)	*E*corr (V_SCE_)	*ΔE* (V)	*E*t (V)
W20N0.5	2632.29	585	1949.72	4.920 × 10^−7^	−0.660	1.166	1.615
W5	2448.36	458	1435.45	1.133 × 10^−7^	−0.586	1.262	>2.5

**Table 3:** Standard electrode potential of main elements in RHEA.

Anodic Chemical Reaction	Standard Electrode Potential (V)
Ti(s) → Ti^2+^ + 2e	−1.63
Zr(s) → Zr^4+^ + 4e	−1.45
V(s) → V^2+^ + 2e	−1.175
Ta(s) → Ta^3+^ + 3e	−0.6
W(s) → W^3+^ + 3e	0.1

## Data Availability

The data presented in this study are available on request from the corresponding author.
